# Optimizing Cooperative Community Hospital Selection for Post-Discharge Care with NSGA-II Algorithm

**DOI:** 10.3390/healthcare14030372

**Published:** 2026-02-02

**Authors:** Zhenli Wu, Yunxuan Li, Xin Lu

**Affiliations:** College of Systems Engineering, National University of Defense Technology, Changsha 410073, China; wuzhenli@nudt.edu.cn (Z.W.); liyunxuan@nudt.edu.cn (Y.L.)

**Keywords:** post-discharge care, follow-up service, medical resource allocation, multi-objective mixed-integer programming, NSGA-II algorithm

## Abstract

**Background**: With the growing emphasis on full-process disease management, efficient post-discharge care has become increasingly critical. Although prior studies have examined follow-up services, resource allocation, and facility location in primary healthcare, model-based optimization of collaborative frameworks between comprehensive hospitals and primary care systems remains limited. **Methods**: We study a cooperative community hospital selection problem involving contractual cooperation, patient engagement, and follow-up resource allocation. A multi-objective mixed-integer programming model is developed to maximize patient accessibility and minimize total hospital costs, and an NSGA-II-based heuristic is proposed for solution generation. A real-world case study using data from a comprehensive hospital in Chengdu, China, is conducted. **Results**: The proposed approach produces a Pareto set that quantifies the accessibility–cost trade-off and reveals a knee region with diminishing returns: moderate expansion of cooperating providers substantially improves accessibility, whereas further expansion yields limited additional gains while increasing hospital cost. Sensitivity analyses indicate that cost-related parameters and follow-up frequencies are key drivers of the trade-off. **Conclusions**: The proposed optimization framework serves as an implementable decision aid for designing hospital–primary care collaboration for post-discharge follow-up: it supports partner selection and capacity planning and indicates levers to improve performance.

## 1. Introduction

Follow-up service has been shown to provide discharged patients with timely diagnosis, treatment and rehabilitation guidance through regular examinations and observations [1,2]. The benefits of this service include enhanced quality of life and health status of discharged patients [3,4], reduced likelihood of re-admission [5,6], and decreased economic burden associated with unnecessary hospital stays. Some studies also report lower post-discharge mortality in certain patient groups, although results vary by population and intervention type [2,7].

For hospitals, follow-up visits help decrease re-admission rates, improving bed availability and enabling the prioritization of acute and critical care [8,9]. Additionally, these visits allow for the collection and analysis of essential patient data, aiding in the evaluation of diagnostic and treatment protocols and the establishment of more accurate standards in the future [10]. Thus, follow-up visits are crucial for both healthcare professionals and patients.

On a global scale, follow-up visits have been incorporated into chronic disease management and post-acute treatment rehabilitation in various healthcare systems. In developed countries, the responsibility for follow-up has been progressively delegated to primary care through a system of hierarchical diagnosis and treatment [11]. Concurrently, the implementation of remote monitoring devices (e.g., wearable electrocardiographs) has driven the expansion of telephone and video follow-up [12,13]. In addition, the combination of electronic health records (EHRs) and artificial intelligence technology supports risk stratification and precise intervention [14,15].

However, several challenges persist in the delivery of follow-up care. First, a significant gap exists between the high demand for follow-up visits and the limited healthcare capacity of hospitals, leading to service disruptions. Healthcare organizations tend to prioritize clinical needs when allocating resources, often overlooking patients’ non-clinical needs. To provide continuous support and better address these needs, healthcare organizations must shift from a supply-driven to a demand-driven service model [11]. Additionally, the healthcare system should increase financial and human resource investments in primary care, expanding the roles of nurses and medical assistants to manage preventive care and chronic disease coaching, thereby meeting the growing expectations for healthcare services [12].

Second, teleconsultations may not fully replace in-person follow-up visits, particularly when physical examinations and comprehensive evaluations are necessary, as they can increase the risk of misdiagnosis and sub-optimal interventions. Moreover, teleconsultations may exacerbate healthcare access disparities among elderly patients. In contrast, face-to-face follow-ups, while often more time-consuming, have been shown to be superior in strengthening doctor–patient relationships, enhancing patient education, and improving consultation quality. Notably, most studies focus on follow-up visits conducted at designated healthcare facilities rather than in patients’ homes, which may influence the naturalness and effectiveness of communication [13].

Finally, geographic disparities in healthcare access must be addressed. Rural residents often face poorer health outcomes and limited access to medical services [14], while traffic congestion in urban areas increases travel times and reduces service availability [16]. If left unresolved, these barriers could contribute to preventable hospital re-admissions, higher patient mortality, and additional financial strain on healthcare systems.

These challenges indicate that post-discharge care requires systematic improvement. Due to the characteristics of high frequency, long duration, and scattered distribution of patients, although general hospitals bear substantial follow-up demand, they face difficulties in independently completing all follow-up tasks in a sustained and stable manner under the combined effects of resource constraints and insufficient geographic accessibility. Meanwhile, relying solely on remote follow-up has limited effectiveness for certain populations and complex care scenarios. In contrast, primary healthcare systems are closer to communities and are better suited to undertake routine follow-up and continuous health management, thereby enhancing accessibility and adherence [17]. However, primary healthcare systems exhibit variability in service capacity and quality and often require hospitals to provide clinical guidance, training support, and information sharing to ensure follow-up quality and risk referral [18]. Therefore, it is necessary to establish a collaborative follow-up mechanism between general hospitals and primary healthcare systems to integrate hospital-level clinical supervision with community-level follow-up services.

It is important to emphasize that such collaboration must be operationalized through executable contractual arrangements. In practice, collaboration between general hospitals and primary healthcare systems often operates under hierarchical governance structures and involves explicit costs and responsibilities, including contracting costs, hospital capacity-building investments, quality responsibilities, and patient referral and information-sharing mechanisms [19]. Consequently, the implementation of collaboration naturally transforms into a joint decision-making optimization problem that trades off patient accessibility against the hospital’s total cost.

Our main contributions are as follows:

(1) Problem setting and decision layers. While many healthcare studies treat the service network as given and optimize only allocation or routing, we study a post-discharge general hospital–primary care collaboration problem. Collaboration is not assumed; instead, it is endogenized as executable contracting decisions under a hierarchical governance structure, which couples (i) partner selection at two tiers, (ii) contract activation, and (iii) downstream patient assignment.

(2) Operationalizable cost and capacity integration. Beyond the common facility-opening and service cost structure, our model explicitly incorporates (i) contracting overheads, (ii) hospital-side capacity-building investments transferred to cooperating providers, and (iii) modality-dependent workload and costs (facility-based vs. home follow-ups) under provider capacity constraints. This yields a multi-objective mixed-integer program that captures implementable responsibilities and budget implications rather than a purely accessibility–cost trade-off.

(3) Constraint-aware NSGA-II design, driven by hierarchy and proximity logic. Instead of applying a generic binary encoding with penalty terms, we design a problem-specific chromosome that jointly represents hospital selections with embedded hierarchical relations, and we induce allocations through a proximity-based assignment consistent with practice. We further introduce a consistency repair mechanism that eliminates selected-but-unused facilities and enforces hierarchical contracting logic, which significantly improves search efficiency in this constrained mixed-integer setting.

(4) Managerial insights tied to collaboration levers. Using real data from a Chengdu general hospital, we not only report Pareto fronts but also interpret trade-offs through collaboration levers that are actionable for hospital managers (e.g., contracting overhead, capacity-building cost, and follow-up frequencies).

The remainder of this article is structured as follows: Section 2 provides a literature review. Section 3 formally defines the problem, introduces the model formulation, presents a heuristic approach, and describes the experimental setting. Section 4 reports and discusses the results. Section 5 outlines the main limitations and directions for future research, and Section 6 concludes with key findings.

## 2. Literature Review

This study proposes an optimization framework for collaborative networks for post-discharge care between large general hospitals and primary care institutions. To motivate this paradigm and position our contribution, we synthesize related work along three streams: (i) post-discharge care within primary healthcare systems, (ii) hospital selection and patient allocation models for post-discharge care, and (iii) optimization-based models for collaborative healthcare networks. Subsequently, we conduct a summary to identify the research gaps that this study aims to address.

### 2.1. Post-Discharge Care Within Primary Healthcare Systems

A substantial body of empirical and clinical research supports the effectiveness of community-based follow-up after discharge and highlights the role of primary healthcare providers in improving continuity of care. Herrin et al. [20] suggest that follow-up incentive policies may be more effective when they target a broader care system that includes primary care and nursing homes rather than relying solely on large hospitals. Toth et al. [21] report that a higher density of primary care providers is associated with a higher probability of receiving follow-up within 14 days after discharge. From a patient-needs perspective, Castro et al. [22] show that ICU-discharged patients often require longer-term services (e.g., rehabilitation training and therapy) that are more accessible at the community level.

However, this stream is primarily effectiveness and policy-oriented. While it clarifies why community follow-up matters and what services are needed, it often leaves the operational question under-specified: given heterogeneous patient needs and limited hospital capacity, how should follow-up demand be systematically distributed across providers? In addition, the literature distinguishes follow-up modalities (e.g., face-to-face vs. phone follow-ups) and their impacts [6,23], but typically does not translate modality differences into an integrated, capacity-constrained planning model at the network level—a gap directly related to our collaborative networks design.

### 2.2. Hospital Selection and Patient Allocation Models for Post-Discharge Care

Hospital selection in collaborative contexts is closely related to facility location and patient allocation models. From a network design perspective, Rahman and Smith [24] address rural facility locations using a maximum coverage model with heuristics, while Smith et al. [25] propose a mixed-integer model for sustainable community healthcare planning considering hierarchical site selection. Veenstra et al. [26] integrate facility location with vehicle routing in healthcare logistics. These studies provide methodological foundations for selecting service nodes and designing delivery networks.

From the patient perspective, travel distances and out-of-pocket expenses are major determinants of utilization and adherence. Li et al. [27] identify cost as a barrier to follow-up participation among gastric cancer survivors, noting that follow-up expenses may not be covered by insurance and that rural patients face additional accommodation and time costs when traveling to urban providers. Similar access concerns are reported for patients requiring timely outpatient follow-up after myocardial infarction [13]. Cote et al. [14,28] incorporate patient treatment, accommodation, and transportation costs in mixed-integer programming models.

In the context of patient allocation to hospitals, Harper et al. [29] incorporate capacity and demand fluctuations using a stochastic geographical simulation model to study patient travel across multiple hospitals. Patrick et al. [30] formulate dynamic patient assignment with priority classes using a Markov decision process and approximate dynamic programming. Yuan et al. [31] propose heuristic assignment rules that combine priority ranking and distance considerations. These works demonstrate that optimization can meaningfully improve access and efficiency when demand and capacity constraints are present.

Nevertheless, existing models are often tailored to either hospital-centric allocation or single-service operational planning, and they may not explicitly model the collaborative structure and decision layers required in post-discharge care networks. In particular, many studies do not simultaneously decide (i) which healthcare providers should participate (network design), (ii) how much follow-up workload each provider should undertake under capacity limits, and (iii) how heterogeneous follow-up modalities (e.g., outpatient visits vs. home visits) translate into differentiated resource consumption and costs. These gaps motivate the integrated optimization paradigm proposed in this study.

### 2.3. Optimization-Based Models for Collaborative Healthcare Networks

Recent optimization-based studies on collaborative healthcare networks span strategic network redesign, technology-enabled coordination, and network-level performance improvement. Wang et al. [32] optimize hierarchical facility layouts using multi-source data and location–allocation modeling, while Mitropoulos et al. [33] couple system dynamics with two-stage stochastic optimization to redesign a primary care network under endogenous-demand scenarios. At the operational level, Gong and Tang [34] formulate mixed-integer (stochastic) resource allocation for a hospital–community–family edge-network intervention system. In emergency contexts, Chen et al. [35] integrate deep learning prediction with multi-objective optimization to coordinate multi-echelon resource allocation, and Soltani et al. [36] model collaboration via cooperative games and robust planning for IoT-enabled emergency transportation. Beyond these settings, Liu et al. [37] show that coordinated care outcomes depend on network-wide interactions and propose a network-level approach to improve itinerary completion.

Nevertheless, these studies leave gaps for discharge care planning between large general hospitals and primary care institutions. Existing models either emphasize spatial redesign and long-run service configuration [32,33] or focus on technology- or uncertainty-driven coordination in chronic disease management and emergency response [34,35,36], which differ from routine, capacity-constrained post-discharge follow-up operations. Moreover, while network-level coordination is highlighted [37], prior work is still lacking in specific post-discharge scenarios and the selection of coordinating network partners, which is why this study proposes a comprehensive optimization paradigm.

## 3. Materials and Methods

### 3.1. Problem Statement and Model Formulation

As the largest hospital in Chengdu, a major metropolitan area in southwest China, the General Hospital serves a vast number of patients discharged after receiving treatment for conditions such as coronary heart disease, myocardial infarction, and aortic valve disorders. These patients generate a significant demand for follow-up care. As a critical component of patient management and a cornerstone of healthcare continuity, in-person (offline) follow-up visits have been shown—both in academic research [16,38] and in practice—to yield superior outcomes.

However, the local family doctor system is still in its early stages and faces several challenges in providing follow-up care. These challenges include lower patient trust in family doctors and the lack of an effective information-sharing mechanism between hospitals and family physicians [39]. Additionally, given the large number of discharged patients dispersed across the city, the availability of medical staff remains limited. To address this issue, the General Hospital plans to collaborate with regional medical centers (RMCs) and primary health centers (PHCs) for follow-up care, leveraging the ongoing development of medical consortia.

#### 3.1.1. Study Area and Data Collection

**Figure 1:**
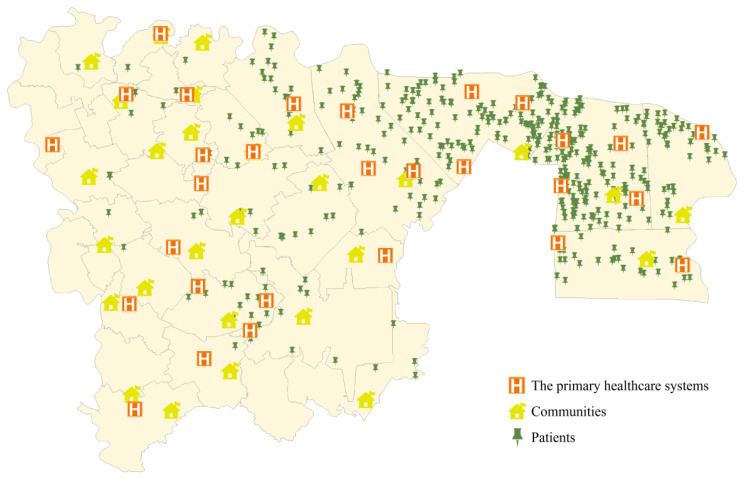
Illustration of Wuhou District, with the location of patients, communities and hospitals.

Wuhou District is located in the southwestern part of Chengdu City, Sichuan Province, with a total area of 75.36 square kilometers. As one of the old five districts of Chengdu, Wuhou District is named after the famous Wuhou Shrine in the district. As of the end of 2023, Wuhou District had a resident population of 1.91 million and 1,324 healthcare facilities. After checking the data on the website of the Chengdu Health Committee by phone, the locations of 29 RMCs and their subordinate PHCs were obtained. The Cardiovascular Disease Unit of the General Hospital has 726 patients distributed within 28 communities in Wuhou District (see Figure 1).

#### 3.1.2. Cooperative Hospital Selection Model

**Figure 2:**
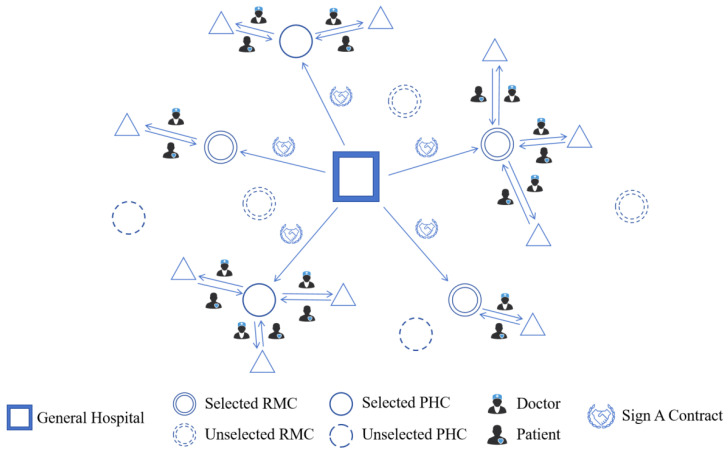
Illustration of hospital follow-up network.

The problem of RMC and PHC selection for follow-up visits is illustrated in Figure 2. The General Hospital contracts and cooperates with some RMCs for one year in the area. These RMCs, as well as their subordinate PHCs, then carry out follow-up service for the General Hospital’s discharged patients in the area.

Formally speaking, the set $I_{1}$ and $I_{2}$ is used to denote all the RMCs and all their subordinate PHCs, respectively, where an RMC or a PHC is indexed by *i*. It costs $C_{1}$ for the General Hospital to contract with each RMC for one year if it plans to cooperate with an RMC or its subordinate health PHC, as the PHCs do not have independent authority for contracting due to the hierarchical healthcare governance framework. We use the parameter $a_{ii^{\prime }}\;$ to denote the subordinate relationship, and we denote that a PHC $i\in I_{2}$ is subordinate to an RMC $i^{\prime }\in I_{1}$. For the sake of modeling, we uniformly apply $a_{ii}=1$ to represent that an RMC is the subordinate of itself, and $a_{ii^{\prime }}=0$ ($i\neq i^{\prime }$) if RMCs *i* and $i^{\prime }$ have no subordinate relationship.

All the demand points of discharged patients are included in the set *J*, where a demand point is indexed by *j*. The average number of discharged patients per year at demand point *j*, estimated by historical data, is $n_{j}$. The patients are classified into |K| types according to the different follow-up services they need, which can be corresponded to clinical risk stratification or disease stage. We denote the proportion of Type k∈K patients at demand point j∈J as $p_{j}^{k}$. Each Type k∈K patient has a requirement of $F_{k}^{1}$ (resp. $F_{k}^{2}$) number of follow-up services at RMC/PHC (resp. his home).

The follow-up frequencies $F_{k}^{1}$ and $F_{k}^{2}$ are treated as exogenous planning parameters because, in many healthcare systems, follow-up schedules are standardized by clinical pathways, chronic disease management packages, or post-discharge protocols over a given planning horizon. To reflect heterogeneity, frequencies are specified by patient type |K|, rather than assuming a single uniform frequency. We acknowledge that individual-level schedules may vary; therefore, we evaluate robustness by varying $F_{k}^{1}$ and $F_{k}^{2}$ in sensitivity analysis.

The General Hospital provides some follow-up service capacity for the cooperating RMCs and PHCs, e.g., medical suppliers and training. It costs $C_{2}$ to provide unit service capacity.

It takes an average service capacity of $e_{k}^{1}$ (resp. $e_{k}^{2}$) for each Type k∈K patient’s follow-up service at the RMC (resp. his home). It costs $c_{ijk}^{1}$ (resp. $c_{ijk}^{2}$) for the RMC/PHC $i\in I_{1}\cup I_{2}$ to provide follow-up service for a Type k∈K patient of demand point j∈J at the RMC (resp. his home) every time. All the above costs are ultimately attributed to the General Hospital. On the other hand, it also costs $h_{ijk}^{1}$ (resp. $h_{ijk}^{2}$) for each Type k∈K patient of demand point j∈J to have a follow-up service at the RMC (resp. his home) provided by RMC/PHC $i\in I_{1}\cup I_{2}$ every time.

The community demand point needs to be assigned to each RMC/PHC where the follow-up work will be carried out. Let $d_{ij}$ be the travel distance between an RMC/PHC $i\in I_{1}\cup I_{2}$ and a community demand point j∈J. For the convenience of both patients and medical staff, each demand point of discharged patients is allocated to the RMC/PHC closest to it, i.e., the principle of proximity.

This problem considers both social benefits (patient accessibility) and economic benefits (total cost of follow-up) and is aimed at minimizing the cost of all the discharged patients and the cost of the General Hospital, with decisions on the selection of the RMCs/PHCs ($x_{i}$), the contract ($y_{i}$), the patients allocation ($z_{ij}$), and the follow-up service capacity provision ($w_{i}$).

All the notations are summarized in Table 1.

By employing the aforementioned notational framework, the research problem is formulated as the following multi-objective mixed-integer linear programming model. For the General Hospital, there is a desire for greater patient accessibility in the follow-up service. Therefore, the cost of follow-up services at the hospital for patients from each community is minimized as the objective function, that is,

(1) $$f_{1}=\sum_{k\in K}\sum_{i\in I_{1}\cup I_{2},j\in J}\left(F_{k}^{1}h_{ijk}^{1}+F_{k}^{2}h_{ijk}^{2}\right)n_{j}p_{j}^{k}z_{ij}$$

At the same time, the General Hospital needs to minimize the total cost of the whole follow-up service, including the contracting cost, the cost of follow-up services and the cost of follow-up service capacity, that is,

(2) $$f_{2}=C_{1}\sum_{i\in I_{1}}y_{i}+\sum_{k\in K}\sum_{i\in I_{1}\cup I_{2},j\in J}\left(F_{k}^{1}c_{ijk}^{1}+F_{k}^{2}c_{ijk}^{2}\right)n_{j}p_{j}^{k}z_{ij}+\sum_{k\in K}\sum_{i\in I_{1}\cup I_{2},j\in J}C_{2}\left(F_{k}^{1}e_{k}^{1}+F_{k}^{2}e_{k}^{2}\right)n_{j}p_{j}^{k}z_{ij}$$

Linear cost components are adopted for interpretability and data availability in tactical planning. Over the typical operating range of follow-up programs, transportation costs can be reasonably approximated as proportional to travel distance, which enables transparent parameterization and reproducible optimization. We acknowledge potential nonlinearities (e.g., overtime, batch dispatching, or congestion effects). To mitigate this limitation, we test key unit-cost parameters in sensitivity analysis, and the model can be extended to piecewise-linear or convex cost functions when more granular cost data become available.

For the constraints, each community demand point needs to be assigned to exactly one RMC/PHC for follow-up services, that is,

(3) $$\sum_{i\in I_{1}\cup I_{2}}z_{ij}=1,\;\forall j\in J$$

The RMCs/PHCs providing follow-up services need to establish partnerships with the General Hospital, that is,

(4) $$x_{i}-z_{ij}\geq 0,\;\forall i\in I_{1}\cup I_{2},j\in J$$

The General Hospital, in cooperation with the RMCs/PHCs, must first contract with RMCs, that is,

(5) $$y_{i}-a_{ii^{\prime }}x_{i^{\prime }}\geq 0,\;\forall i\in I_{1},i^{\prime }\in I_{1}\cup I_{2}$$

The number of demand points followed up by each RMC/PHC for one year does not exceed the maximum annual service capacity of the institution, that is,

(6) $$\sum_{k\in K}\sum_{j\in J}\left(e_{k}^{1}F_{k}^{1}+e_{k}^{2}F_{k}^{2}\right)n_{j}p_{j}^{k}z_{ij}\leq w_{i},\;\forall i\in I_{1}\cup I_{2}$$

In our model, proximity is captured through distance-increasing cost terms: the patient-side travel cost $h_{ijk}^{1}$ and the home-visit service cost $c_{ijk}^{2}$ are increasing functions of $d_{ij}$ (discussed in Table 2 and Table 3). Therefore, minimizing $f_{1}$ and $f_{2}$ naturally favors allocating each demand point to nearby hospitals; when the nearest hospital becomes saturated, constraint (6) forces a shift to the next-nearest feasible hospital, which is consistent with practical overflow and then referral operations.

### 3.2. Algorithm and Experimental Setting

The NSGA-II algorithm was selected for this study due to its demonstrated advantages in addressing multi-objective optimization problems: (1) its non-dominated sorting mechanism effectively maintains solution diversity across the objective space; (2) the crowding distance operator facilitates uniform distribution along the Pareto front, thereby preventing premature convergence to local optima; and (3) the algorithm exhibits superior convergence characteristics and robustness when handling mixed-integer decision variables, which aligns well with the structural requirements of the proposed model. To further substantiate its appropriateness for this application, a comprehensive comparative analysis against multiple benchmark algorithms is presented in Section 4.2.

**Figure 3:**
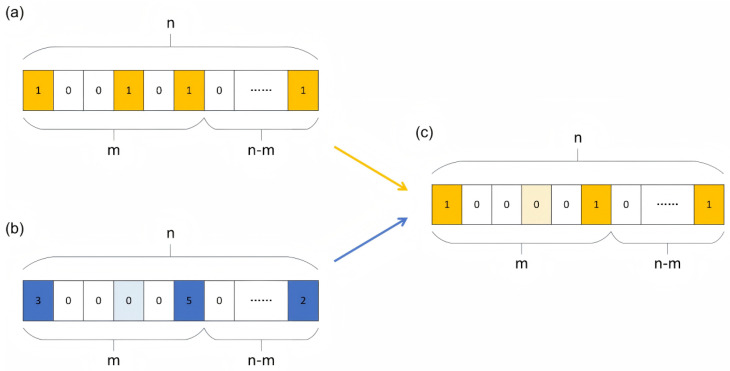
The construction of chromosomes, (**a**) Initial chromosome matrix; (**b**) Community demand point allocation matrix; (**c**) Chromosome correction matrix.

In this algorithm, each chromosome represents a scheme for selecting cooperative follow-up facilities within RMCs or PHCs. The construction of chromosomes is achieved through three matrices: (a) the initial chromosome matrix, (b) the community demand point allocation matrix, and (c) the chromosome correction matrix. The procedures for representing these matrices are described as follows:

(1) Initial chromosome matrix: The initial chromosome is represented as a 1×n matrix, as illustrated in Figure 3a, where *n* denotes the total number of candidate facilities, comprising both RMCs and their associated PHCs. Specifically, the first *m* elements correspond to PHCs, while the subsequent n−m elements represent RMCs, with predetermined hierarchical relationships established between them. Each element assumes a binary value of 0 or 1, where 0 signifies facility exclusion and 1 indicates facility selection. For instance, Figure 3a depicts a configuration where the 1st, 4th, 6th, …, and *n*-th facilities are selected. The initial selection is performed stochastically to ensure population diversity.

(2) Community demand point allocation matrix: This matrix maintains the 1×n dimensional structure, as presented in Figure 3b. In contrast to the binary chromosome matrix, each element herein quantifies the number of community demand points allocated to the corresponding selected facility based on the principle of spatial proximity. For example, Figure 3b demonstrates that the 1st, 4th, 6th, …, and *n*-th facilities are assigned 3, 0, 5, …, and 2 patients, respectively.

(3) Chromosome correction matrix: This matrix, depicted in Figure 3c, rectifies potential allocation inconsistencies that emerge when selected facilities receive zero demand point assignments, as exemplified in Figure 3b. Such scenarios indicate sub-optimal facility selection. To address this issue, facilities with null allocations are systematically deselected, and the chromosome is correspondingly modified to ensure solution feasibility and consistency. For instance, given that the 4th facility in Figure 3b receives no patient allocation, it is subsequently excluded from the solution, as reflected in Figure 3c.

Upon completion of these three procedural steps, the chromosome-construction process is finalized, yielding a valid and structured solution representation for the optimization problem.

The parameters of the multi-objective genetic algorithm NSGA-II must be adapted to suit the particular problem at hand. In this particular instance, the population size was set to 100, the number of iterations to 500, the number of individuals involved in the tournament to 100, the probability of tournament selection to 0.9, the crossover parameter to 2, and the variation parameter to 5. This configuration was arrived at through a series of experiments.

In the context of this planning project for the General Hospital, certain parameter values (see Table 2) were determined through predictive analysis based on established reality (see Table 3).

## 4. Results

### 4.1. Performance of the Model

**Figure 4:**
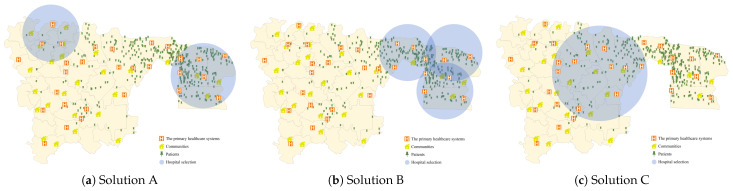
Illustration of the comparative solutions. (**a**) Solution A. (**b**) Solution B. (**c**) Solution C.

To establish a robust comparative analytical framework, we strategically delineated three distinct selection approaches: Solution A comprised 10 hospitals selected from two geographically distal regions with maximal inter-regional distance (shown in Figure 4a); Solution B encompassed 10 hospitals drawn from the region exhibiting the highest patient population density (shown in Figure 4b); and Solution C included 10 hospitals situated within the central region (shown in Figure 4c).

**Figure 5:**
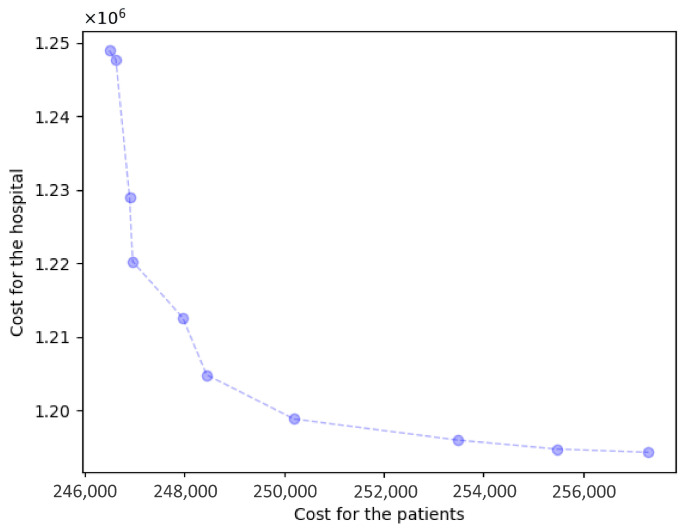
Pareto frontier surface.

**Figure 6:**
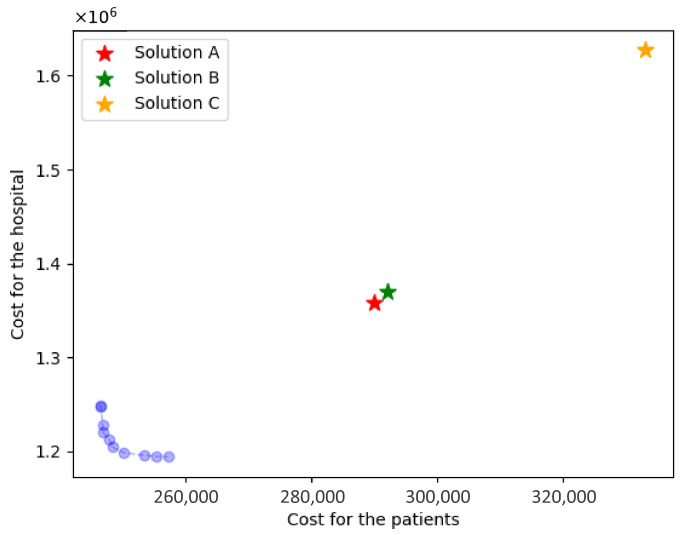
Comparison of Pareto frontier surface with Solutions A, B, and C.

The cooperative hospital selection model obtains 10 Pareto solutions; the results comparison is shown in Table 4. Figure 5 elucidates the Pareto frontier surface, while Figure 6 provides a comparative visualization of the Pareto solutions relative to Solutions A, B, and C. We found that as the number of selected hospitals increases, the patient costs decrease while the hospital costs increase, which signifies that patient accessibility is high, with abundant healthcare service resources. However, when the number of selected hospitals exceeds 15, there is no significant improvement in patient accessibility, while the hospital costs surge dramatically to 1.2 million or more. Conversely, when the number of selected hospitals is below 13, even a slight reduction in hospital costs leads to a sharp increase in patient costs to 250 thousand. This indicates that when the resources invested by the General Hospital are limited, patients encounter great challenges in accessing healthcare services.

**Figure 7:**
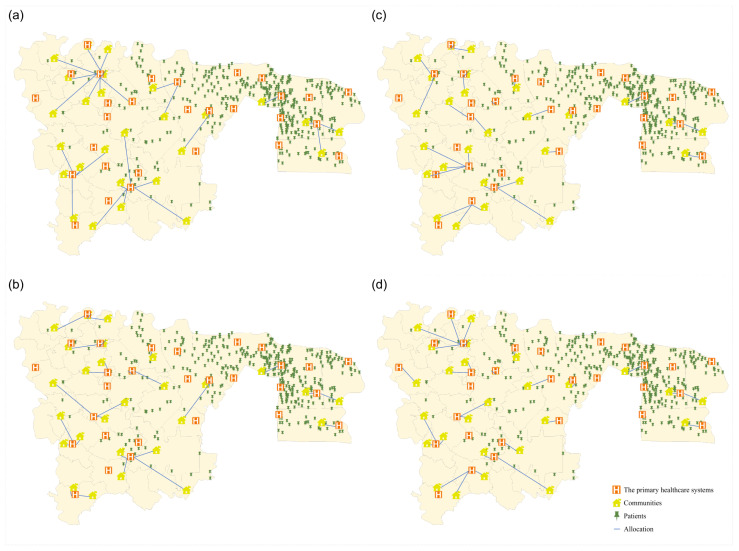
Illustration of four Pareto solutions. (**a**) Solution 1; (**b**) Solution 4; (**c**) Solution 7; (**d**) Solution 10.

The following four solutions were illustrated: Solutions 1, 4, 7, and 10, corresponding to the selected hospital counts of 7, 13, 15, and 16, respectively, as depicted in Figure 7a–d, which offer an intuitive depiction of the allocation of patients across the 28 communities and the selected cooperative hospitals under various decision conditions. It has been observed that the majority of patients in Wuhou District are concentrated within four communities on the right side. Given the service capacity of hospitals, it is estimated that two to three hospitals would be required to handle the follow-up care for these patients. The remaining small number of patients is distributed across 24 communities on the left side. Due to the vast area and sparse population, the establishment of more hospitals is necessary to serve these patients, thus avoiding incurring greater costs for patients or doctors when traveling across districts. The distribution relationship between hospitals and patients is radial in nature, primarily stemming from the principle of proximity in patient healthcare access.

The Pareto set in Figure 5 provides a direct decision aid for hospital managers when forming post-discharge collaboration with primary care providers. In practice, selecting a solution near the “knee” of the frontier (e.g., Solution 5) yields substantial improvements in patient accessibility, with a moderate increase in the hospital’s total cost, whereas moving further toward extreme access-focused solutions produces only marginal accessibility gains but requires disproportionately higher contracting and capacity-building expenditures. Operationally, each Pareto solution corresponds to an implementable contracting plan (which PHCs are activated under each RMC) and patient allocation, which can be translated into follow-up workload plans and staffing requirements for facility-based and home follow-ups. From a clinical process perspective, improved geographic access and feasible workload allocation support timely follow-up visits after discharge, thereby strengthening continuity of care and reducing missed follow-ups; this is the main pathway through which the proposed collaboration design can potentially improve downstream clinical outcomes.

### 4.2. Benchmarking for Algorithm Selection

To assess the suitability and practical performance of NSGA-II in our mixed-integer multi-objective context, we benchmarked it against two representative algorithms: SPEA2 and MOEA/D. SPEA2 promotes diversity and elitism through an external archive and strength-based fitness assignment, whereas MOEA/D decomposes the multi-objective problem into a set of scalar subproblems and searches via neighborhood collaboration.

Each algorithm is executed under the same computational budget. To account for stochasticity, we performed S=20 independent runs with different random seeds for each algorithm. For every run, we evaluated the final non-dominated set produced by the algorithm. Since the objectives $f_{1}$ and $f_{2}$ differ in scale, we applied min–max normalization to ensure comparability for distance/area-based indicators.

We report three widely used indicators (computed in the normalized objective space):

- HV (Hypervolume): Measured with reference point r=(1.2,1.2); a larger HV indicates better combined convergence and coverage.
- IGD (Inverted Generational Distance): Average distance from a reference front to the obtained set; a smaller IGD indicates better proximity to the reference front.
- Spacing: Measures the uniformity of spacing among solutions; a smaller value indicates a more even distribution.

As the true Pareto front is unavailable, we constructed a pooled reference set using the union reference front. Specifically, we merged all final non-dominated sets from all algorithms and all seeds, and we filtered the merged set again to retain only non-dominated points.

Table 5 reports the results over S=20 independent runs and serves as a post hoc validation of our earlier choice of NSGA-II as the solution algorithm. Under the same evaluation budget, NSGA-II achieves the highest HV and the lowest IGD, indicating the strongest overall performance in terms of convergence toward the pooled reference front and coverage of the objective space, which are the primary criteria in our study. Although SPEA2 yields the smallest Spacing (i.e., more regular distributions) and a comparable IGD, its lower HV suggests weaker coverage, particularly near extreme trade-off regions. MOEA/D attains reasonable HV and uniformity, but its noticeably larger IGD implies insufficient convergence. Overall, these observations are consistent with our decision to use NSGA-II in the main experiments, while SPEA2 and MOEA/D provide complementary references emphasizing distribution regularity and alternative search dynamics, respectively.

### 4.3. Sensitivity Analyses

To derive managerial insights for selecting cooperative community hospitals for post-discharge care, we performed scenario-based sensitivity analyses on four key parameters: the contracting cost $C_{1}$, the unit service-capacity cost $C_{2}$, and the follow-up frequencies $F_{k}^{1}$ and $F_{k}^{2}$.

We evaluate the Pareto sets obtained under each parameter level using the hypervolume (HV) indicator. For a multi-objective minimization problem, the HV of a non-dominated set *P* with respect to a reference point r is defined as the Lebesgue measure of the portion of the objective space dominated by *P* and bounded by r:

(7) $$HV\left(P;r\right)=\lambda \;\left(\underset{z\in P}{⋃}\left[z,r\right]\right).$$

Here, [z,r] denotes the axis-aligned hyper-rectangle spanned by a solution objective vector z∈P and the reference point r, and λ(·) is the Lebesgue measure. To ensure strict comparability across scenarios, we used a single global reference point determined from the aggregated outcomes of all scenarios. A larger HV indicates a Pareto set with better overall convergence and diversity.

Table 6, Table 7, Table 8 and Table 9 summarize the results. Beyond reporting directional changes, we interpret why certain parameters dominate system behavior by linking the observed shifts of the Pareto frontiers to the model structure: (i) cost parameters enter the hospital-cost objective directly and therefore shift the feasible trade-off surface; and (ii) follow-up frequencies affect both the required service volume and the feasibility of proximity-based, capacity-constrained assignment, thereby altering not only costs but also the structure of feasible allocations. The baseline scenario is highlighted in bold.

**Figure 8:**
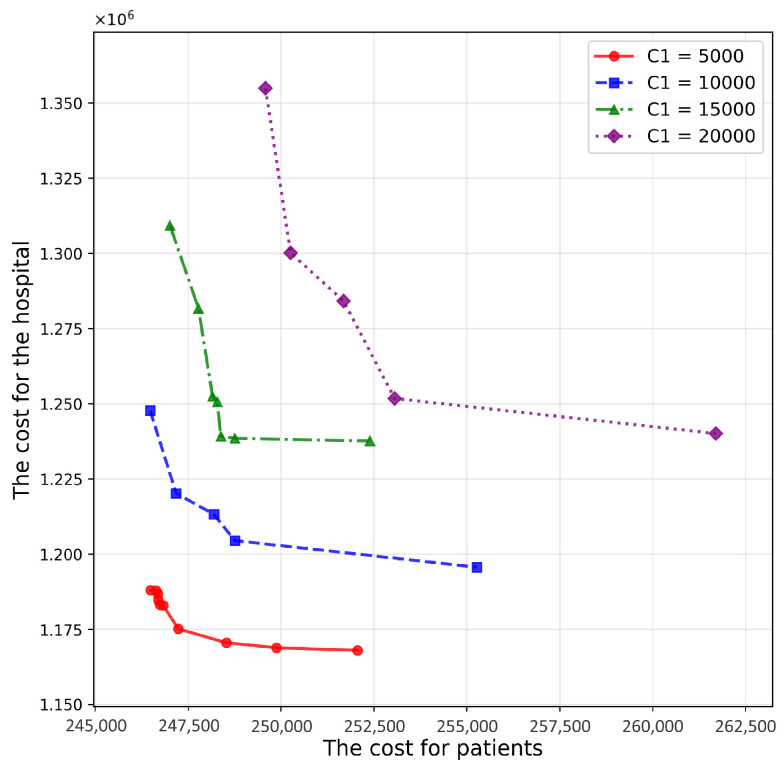
Pareto frontier surfaces for various values of $C_{1}$.

Figure 8 illustrates the Pareto frontier surfaces under different values of $C_{1}$. As $C_{1}$ increases, the Pareto front deteriorates (Table 6), reflecting that higher contracting costs raise the hospital’s total cost and reduce the attractiveness of contracting additional community partners. Mechanistically, $C_{1}$ acts as a fixed overhead associated with establishing and maintaining cooperative relationships; when this overhead is high, the model tends to favor fewer contracted providers unless the accessibility gains are sufficiently large. From a managerial and policy perspective, this finding suggests that reducing contracting costs—e.g., centralized contract administration and targeted subsidies for collaboration setup—can improve the cost–accessibility trade-off without changing clinical follow-up intensity.

**Figure 9:**
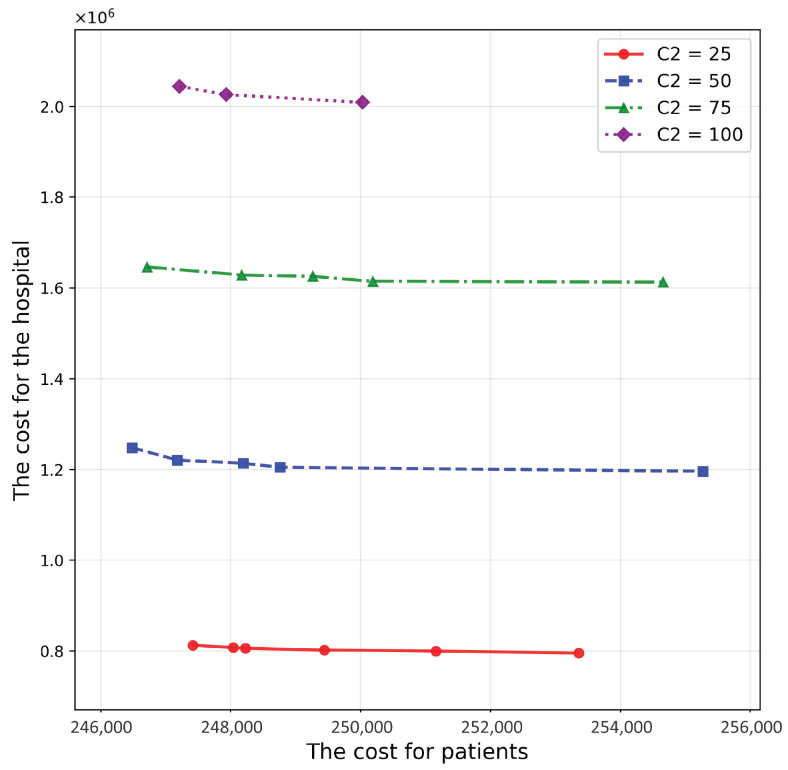
Pareto frontier surfaces for various values of $C_{2}$.

Figure 9 shows the Pareto frontier surfaces for various values of $C_{2}$. The HV decreases markedly as $C_{2}$ rises (Table 7), indicating that unit capacity cost is a dominant driver of the hospital-side objective. This dominance is expected because $C_{2}$ scales the marginal cost of the provisioning follow-up capacity required to serve allocated patients; once proximity-based assignment is enforced, insufficient or expensive capacity forces either (i) additional investment to satisfy capacity constraints or (ii) less favorable allocations that worsen the trade-off. Managerially, interventions that lower the per-unit cost of follow-up capacity—such as scalable training programs, shared clinical protocols, interoperable information systems, and resource pooling across PHCs under an RMC—are likely to yield disproportionate benefits relative to measures that only reduce one-time contracting overhead.

**Figure 10:**
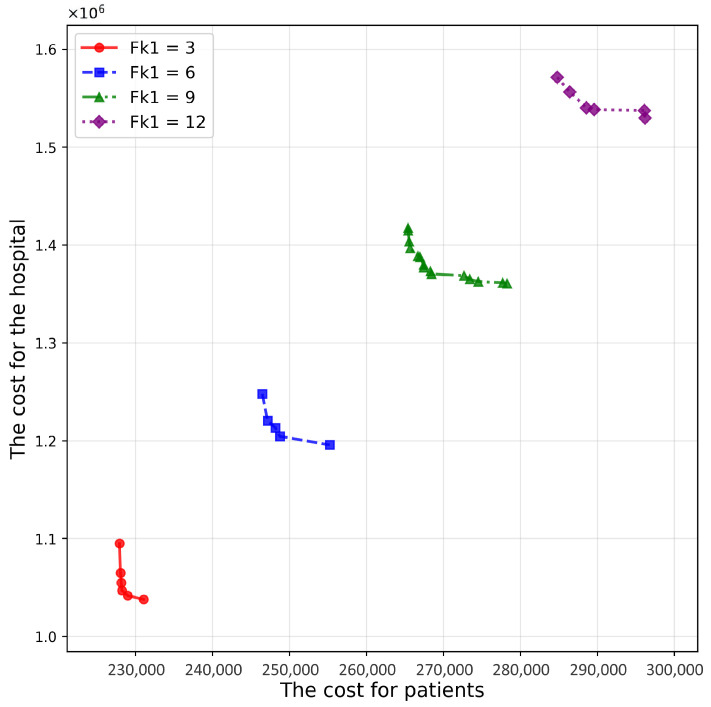
Pareto frontier surfaces for various values of $F_{k}^{1}$.

**Figure 11:**
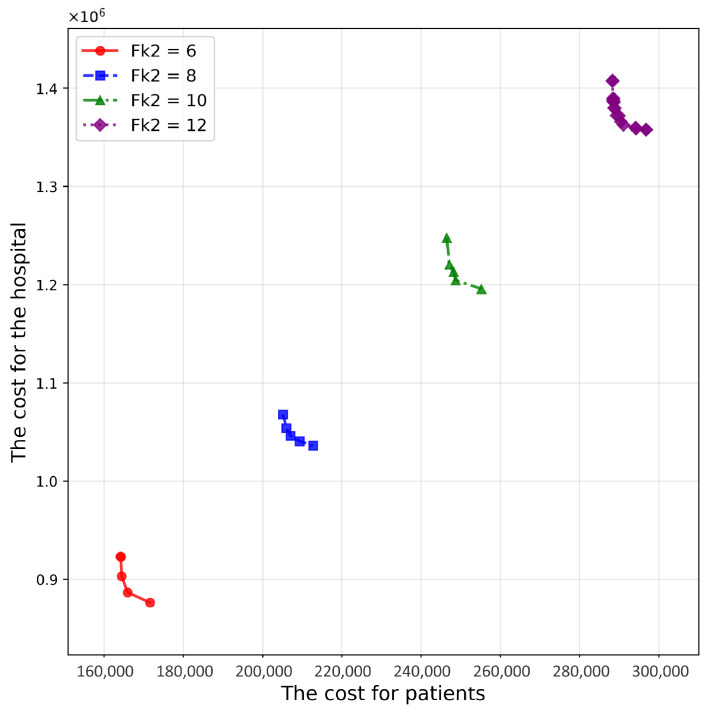
Pareto frontier surfaces for various values of $F_{k}^{2}$.

Figure 10 and Figure 11 present results for different follow-up frequencies, $F_{k}^{1}$ (hospital-based) and $F_{k}^{2}$ (home-/community-based). Increasing either frequency substantially degrades HV (Table 8 and Table 9) and shifts the Pareto front outward because higher follow-up intensity increases service volume and, thus, amplifies both patient- and hospital-side costs. Importantly, frequency parameters also tighten the capacity constraints: higher required visit counts increase the likelihood that nearby providers reach capacity, which in turn changes feasible proximity-based allocations and may necessitate activating additional providers. This explains why follow-up frequencies can dominate system behavior even when cost coefficients remain unchanged.

These results have direct implications for service design. Rather than applying uniform follow-up frequencies, hospitals and RMCs can implement risk-stratified follow-up pathways: allocate higher-frequency follow-up to high-risk patients while using lower-cost modalities (e.g., telephonic or digital check-ins) for stable patients, and reserve in-person encounters for clinical escalation. Such stratification reduces avoidable service volume while preserving accessibility for patients who benefit most from the follow-up service.

Additionally, demographic composition (e.g., a higher proportion of older patients) can increase community/home follow-up intensity in practice, which is consistent with the sensitivity patterns observed for $F_{k}^{2}$. Therefore, capacity planning and contracting strategies should be adjusted in advance in regions with rapid population aging, prioritizing scalable community capacity building and integrated referral and information-sharing mechanisms.

## 5. Limitations and Future Research

This study has several limitations.

Patient choice and behavioral responses. The model adopts a planner-driven assignment of community demand points to selected RMCs/PHCs and captures “proximity preference” indirectly through distance-increasing cost terms. In reality, patients may exercise choice based on perceived quality, waiting time, familiarity, or insurance constraints and may deviate from the assigned provider. Incorporating explicit patient-choice behavior and endogenous demand reallocation is an important extension.

Demand and workload uncertainty. We treat annual discharged demand and follow-up needs as deterministic averages based on historical patterns. In practice, actual demand fluctuations, variations in patient-type mix, and no-show rates can exhibit substantial temporal and spatial heterogeneity, potentially creating capacity bottlenecks or operational inefficiencies. Beyond these immediate challenges, the rapid advancement of informatics and artificial intelligence in biomedical decision-making [40] suggests that our optimization framework should similarly evolve. Specifically, aligning with the emerging paradigm of Biomedical AI—which integrates digital health infrastructure, physical healthcare systems, and biological science—future refinements of our model could incorporate multimodal physiological data streams and enable deeper human-AI collaboration [41]. Such integration would substantially enhance the responsiveness, adaptability, and precision of post-discharge care networks under dynamic real-world conditions.

Data and generalizability. Transferability of parameter values to other regions may require re-estimation of costs, capacities, and clinical pathways. Multi-region validation is left for future research.

## 6. Conclusions

The findings of this study provide significant insights into the optimization of post-discharge care through cooperative hospital selection. Our model demonstrates the trade-off between patient accessibility and hospital costs, highlighting the importance of strategic resource allocation in healthcare systems. The sensitivity analyses further reveal how key parameters influence overall system performance, offering practical guidance for healthcare managers and policymakers.

The proposed NSGA-II-based approach effectively solves the multi-objective optimization problem, generating Pareto-optimal solutions that allow decision-makers to choose based on their specific priorities. The case study in Chengdu validates the applicability of our model in real-world scenarios, demonstrating its potential to improve post-discharge care continuity and reduce healthcare costs.

## Figures and Tables

**Table 1:** Notations.

**Sets**	
$I_{1}$	Set of all the candidate RMCs, indexed by *i*
$I_{2}$	Set of all the subordinate PHCs, indexed by *i*
*J*	Set of community demand points, indexed by *j*
*K*	Set of patient types, indexed by *k*
**Parameters**	
$C_{1}$	Cost of contracting with an RMC for one year
$a_{ii^{\prime }}$	Equals 1 if an RMC/PHC $i^{\prime }\in I_{1}\cup I_{2}$ is subordinated to the RMC $i\in I_{1}$; otherwise, 0. Let $a_{ii}=1$ for any $i\in I_{1}$
$C_{2}$	Cost of providing unit service capacity for the follow-up service at the RMCs/PHCs
$n_{j}$	Average number of discharged patients per year at the demand point j∈J
$p_{j}^{k}$	Proportion of Type k∈K patients at the demand point j∈J
$F_{k}^{1}$	Average number of follow-up services at the RMC/PHC per year for each Type k∈K patient
$F_{k}^{2}$	Average number of follow-up services at home per year for each Type k∈K patient
$e_{k}^{1}$	Average cost of service capacity at the RMC/PHC for each Type k∈K patient’s follow-up service
$e_{k}^{2}$	Average cost of service capacity at home for each Type k∈K patient’s follow-up service
$c_{ijk}^{1}$	Average cost of providing a follow-up service at the hospital for a Type k∈K patient of the demand point j∈J by the RMC/PHC $i\in I_{1}\cup I_{2}$
$c_{ijk}^{2}$	Average cost of providing a follow-up service at home for a Type k∈K patient of the demand point j∈J by the RMC/PHC $i\in I_{1}\cup I_{2}$
$h_{ijk}^{1}$	Average cost of a follow-up service to the RMC/PHC $i\in I_{1}\cup I_{2}$ for a Type k∈K patient from the demand point j∈J
$h_{ijk}^{2}$	Average cost of a follow-up service at home by the RMC/PHC $i\in I_{1}\cup I_{2}$ for a Type k∈K patient of the demand point j∈J
$d_{ij}$	Travel distance between the RMCs/PHCs $i\in I_{1}\cup I_{2}$ and the demand points j∈J
**Decision Variables**	
$x_{i}\in \left\{0,\;1\right\}$	Equals 1 if the General Hospital selects the RMC/PHC *i* to cooperate; otherwise, 0 ($i\in I_{1}\cup I_{2}$)
$y_{i}\in \left\{0,\;1\right\}$	Equals 1 if the General Hospital contracts with the RMC *i*; otherwise, 0 ($i\in I_{1}$)
$z_{ij}$	Equals 1 if the community demand point *j* is followed up by the RMC/PHC *i*; otherwise, 0 ($i\in I_{1}\cup I_{2},\;j\in J$)
$w_{i}$	Maximum follow-up service capacity of the RMCs/PHCs *i* per year ($i\in I_{1}\cup I_{2}$)

**Table 2:** Parameter values and units.

Parameters	Meanings	Values	Units
$C_{1}$	Cost of contracting with an RMC for one year	10,000	currency/year
$C_{2}$	Cost of providing unit service capacity for the follow-up service at the RMCs/PHCs	50	currency/(capacity unit)
$F_{k}^{1}$	Average number of follow-up services at the RMC/PHC per year for each Type k∈K patient	6	visits/(patient·year)
$F_{k}^{2}$	Average number of follow-up services at home per year for each Type k∈K patient	10	visits/(patient·year)
$e_{k}^{1}$	Average cost of service capacity at the RMC/PHC for each Type k∈K patient’s follow-up service	2	capacity units/visit
$e_{k}^{2}$	Average cost of service capacity at home for each Type k∈K patient’s follow-up service	3	capacity units/visit
$c_{ijk}^{1}$	Average cost of providing a follow-up service at the hospital for a Type k∈K patient of the demand point j∈J by the RMC/PHC $i\in I_{1}\cup I_{2}$	80	currency/visit
$c_{ijk}^{2}$	Average cost of providing a follow-up service at home for a Type k∈K patient of the demand point j∈J by the RMC/PHC $i\in I_{1}\cup I_{2}$	$60\cdot d_{ij}$	currency/visit
$h_{ijk}^{1}$	Average cost of a follow-up service to the RMC/PHC $i\in I_{1}\cup I_{2}$ for a Type k∈K patient from the demand point j∈J	$30\cdot d_{ij}$	currency/visit
$h_{ijk}^{2}$	Average cost of a follow-up service at home by the RMC/PHC $i\in I_{1}\cup I_{2}$ for a Type k∈K patient of the demand point j∈J	50	currency/visit

**Table 3:** Rationales for parameter settings.

Parameters	Rationales
$C_{1}$	Contracting/administrative overhead
$C_{2}$	Mainly personnel expenses at the hospital
$F_{k}^{1}$	Less frequent follow-ups
$F_{k}^{2}$	More frequent follow-ups
$e_{k}^{1}$	Number of staff members followed up at the hospital
$e_{k}^{2}$	Number of staff followed up via home visits
$c_{ijk}^{1}$	Equipment costs at the hospital
$c_{ijk}^{2}$	Distance-proportional equipment costs for home visits (the multiplier 60 represents the assumed travel cost per unit distance)
$h_{ijk}^{1}$	Patient-side distance-proportional cost for hospital visits (the multiplier 30 represents the assumed travel cost per unit distance)
$h_{ijk}^{2}$	Patient-side cost for receiving home service

**Table 4:** Results comparison.

Solution	Counts of Selected Hospitals	Cost for Patients	Cost for the Hospital
1	7	257,313.27	1,194,297.59
2	9	255,472.26	1,194,765.01
3	11	253,492.38	1,195,964.90
4	13	250,206.89	1,198,833.75
5	13	248,451.44	1,204,843.01
6	14	247,975.42	1,212,564.85
7	15	246,969.38	1,220,250.66
8	15	246,906.36	1,228,940.93
9	18	246,631.62	1,247,665.54
10	16	246,498.87	1,248,898.04
A	10	289,989.81	1,358,774.19
B	10	292,200.42	1,370,623.31
C	10	333,162.04	1,627,151.88

**Table 5:** Comparison of multi-objective algorithms.

Algorithms	HV (Mean ± Std)	IGD (Mean ± Std)	Spacing (Mean ± Std)
NSGA-II	1.261 ± 0.021	0.046 ± 0.012	0.053 ± 0.019
MOEA/D	1.234 ± 0.025	0.068 ± 0.013	0.024 ± 0.015
SPEA2	1.211 ± 0.018	0.048 ± 0.010	0.019 ± 0.006

**Table 6:** Results for various values of $C_{1}$.

$C_{1}$	Counts of Solutions	Hypervolume Indicator
5000	10	10,733,861,778
**10,000**	**5**	**7,494,105,618**
15,000	7	6,080,704,666
20,000	5	2,273,658,227

Note: The bold row represents the baseline scenario.

**Table 7:** Results for various values of $C_{2}$.

$C_{2}$	Counts of Solutions	Hypervolume Indicator
25	6	39,314,321,447
**50**	**5**	**25,111,012,262**
75	5	15,512,816,436
100	3	6,200,714,578

Note: The bold row represents the baseline scenario.

**Table 8:** Results for various values of $F_{k}^{1}$.

$F_{k}^{1}$	Counts of Solutions	Hypervolume Indicator
3	6	59,865,055,780
**6**	**5**	**33,494,010,118**
9	15	14,151,315,006
12	6	4,305,551,776

Note: The bold row represents the baseline scenario.

**Table 9:** Results for various values of $F_{k}^{2}$.

$F_{k}^{2}$	Counts of Solutions	Hypervolume Indicator
6	5	96,584,057,144
8	5	54,475,285,865
**10**	**5**	**21,018,668,286**
12	17	3,796,794,391

Note: The bold row represents the baseline scenario.

## Data Availability

The aggregated, community-level data supporting the findings of this study are available from the corresponding author upon reasonable request. The original patient-level data are not available due to privacy and confidentiality restrictions. Researchers requesting the aggregated data will be required to sign a data use agreement.

## Abbreviations

The following abbreviations are used in this manuscript:

NSGA-II	Non-dominated Sorting Genetic Algorithm
EHR	Electronic Health Record
RMC	Regional Medical Center
PHC	Primary Health Center
